# Long term outcomes of occipital nerve stimulation

**DOI:** 10.3389/fpain.2023.1054764

**Published:** 2023-03-20

**Authors:** Monique M. Montenegro, Narayan R. Kissoon

**Affiliations:** ^1^ Department of Neurology, Mayo Clinic, Rochester, MN, United States; ^2^ Department of Anesthesiology and Perioperative Medicine, Mayo Clinic, Rochester, MN, United States

**Keywords:** long term outcomes, habituation, occipital nerve stimulation, headache, neuromodulation

## Abstract

**Background:**

Occipital nerve stimulation (ONS) has been investigated as a potential treatment for disabling headaches and has shown promise for disorders such as chronic migraine and cluster headache. Long term outcomes stratified by headache subtype have had limited exploration, and literature on outcomes of this neuromodulatory intervention spanning 2 or more years is scarce.

**Measures:**

We performed a narrative review on long term outcomes with ONS for treatment of headache disorders. We surveyed the available literature for studies that have outcomes for 24 months or greater to see if there is a habituation in response over time. Review of the literature revealed evidence in treatment of occipital neuralgia, chronic migraine, cluster headache, cervicogenic headache, short lasting unilateral neuralgiform headache attacks (SUNHA) and paroxysmal hemicrania. While the term “response” varied per individual study, a total of 17 studies showed outcomes in ONS with long term sustained responses (as defined per this review) in the majority of patients with specific headache types 177/311 (56%). Only 7 studies in total (3 cluster, 1 occipital neuralgia, 1 cervicogenic headache, 1 SUNHA, 1 paroxysmal hemicrania) provided both short-term and long-term responses up to 24 months to ONS. In cluster headache, the majority of patients (64%) were long term responders (as defined per this review) and only a minority of patients 12/62 (19%) had loss of efficacy (e.g., habituation). There was a high number 313/439 (71%) of adverse events per total number of patients in the studies including lead migration, requirements of revision surgery, allergy to surgical materials, infection and intolerable paresthesias.

**Conclusions:**

With the evidence available, the response to ONS was sustained in the majority of patients with cluster headache with low rates of loss of efficacy in this patient population. There was a high percent of adverse events per number of patients in long term follow-up and likely related to the off-label use of leads typically used for spinal cord stimulation. Further longitudinal assessments of outcomes in occipital nerve stimulation with devices labelled for use in peripheral nerve stimulation are needed to evaluate the extent of habituation to treatment in headache.

## Introduction

Occipital nerve stimulation (ONS) was initially reported by Waisbord in 1985 as being able to offer satisfactory treatment for a patient with occipital neuralgia. Since then, the literature has expanded in reports of therapeutic effect for various refractory headache types (1). ONS has been used to treat intractable occipital neuralgia, migraine, cluster headache, SUNHA, hemicrania continua, paroxysmal hemicrania, and/or cervicogenic headache with systematic reviews showing benefit. There is Level I evidence for the use of ONS in the treatment of chronic migraine (2). However, there have been a limited number of articles which have focused on the long-term evaluation of ONS outcomes. Only a selection of these have reported interim follow-up post-implantation, and at the final follow-up period, to assess whether there was any decrease in response to this neuromodulatory intervention.

Habituation from a physiologic perspective is the diminishing response to a particular frequently encountered stimulus. It has been studied in relation to central nervous system learning pathways, motor and sensory function, and its role in other cognitive domains and function (3). Hindlimb flexion of spinal cats have been a commonly employed model system in Spencer et al.'s, Buchwald et al.'s and Wickelgren's studies (4–6). In Spencer et al., they found that the degree of response decrement was inversely related to stimulus intensity. They presented evidence in this study for response decrement and restoration to be neuronally mediated and not properties of other systemic factors such as blood pressure or circulating agents. At the time of those studies, it was also significant to provide support against habituation defined as a type of fatigue, and this was accomplished through evaluating response restoration by an extrastimulus and concluding that this was due a transient facilitation, i.e., dishabituation response (4). Buchwald et al. aimed to determine whether central or peripheral mechanisms were principally responsible for changes in reflex responses. These reflex responses were phasic responses of high amplitude potentials progressively decreased as stimulation was repeated. Ultimately, it was determined that this evidenced habituation, and that habituation was mediated by central changes in the spinal cord pathway (5). Wickelgren determined that spinal interneurons may be responsible for motoneuron habituation. In this study, 2 theories of habituation were evaluated: synaptic depression theory which proposes either decrease transmitter release or decreased sensitivity to the transmitter and inhibitory buildup theories which proposed an increase in either presynaptic or postsynaptic inhibition in the flexor reflex pathways. The latter was supported by findings from this study (6). Notably, Thompson et al. has detailed history of its exploration in a variety of animal spinal and peripheral nerve studies. In studies of sea slugs' gill withdrawal reflex, an example of peripheral habituation was detailed (7).

Human studies in spinal cord stimulation (SCS) have demonstrated that habituation may occur from 3 months post-procedure to 12 months follow up as evidenced by the results of the ACCURATE randomized control trial. Evidence for habituation has been reported to continue beyond 12 months of treatment by D'Souza et al. (8, 9) Studies of long-term efficacy in SCS have shown sustained pain relief up to 24 months following spinal cord stimulation implant (10–12). Based on these findings it has been proposed that patients with sustained pain relief for greater than 24 months with neuromodulation could be considered in “remission” of chronic pain (13). Evidence is limited regarding the presence of a habituation response during ONS for treatment of intractable headache disorders.

## Current evidence in long term ONS

### Methods

In this review, evidence of habituation was investigated by evaluation of the initially documented post-permanent ONS stimulation response (typically 3 months after implantation to allow for recovery from operative pain) and the final recorded long-term response. We had used a minimum follow up of 24 months due to the long-term efficacy studies observed in SCS, proposed operational definitions of chronic pain remission following neuromodulation, and the observation that habituation typically occurred more than 12 months after implantation in prior SCS studies (9–13).

Studies were found using the search terms: occipital nerve stimulation AND long-term outcomes in Pubmed, Medline, Embase, Google Scholar and SCOPUS and then studies with follow up at 24 months were identified and included… Additional articles were identified by searching topically related new journals not found within Pubmed. They were included if they included the following headache types: occipital headache, migraine, cluster headache, other trigeminal autonomic cephalalgias, cervicogenic headache. Only primary sources were included,systematic reviews or narratives were excluded. Poster abstracts were excluded. These studies were included if they classified headache type within the International Classification of Headache Disorders, any edition They were excluded if they did not focus on occipital nerve stimulation specifically (when multiple nerves were stimulated).

### Cluster headache

Long-term clinical outcomes: Cluster headache had the greatest number of long-term studies with 7 studies. ONS was a favorable treatment modality. The primary endpoints were variable but 4 studies looked at ≥50% decreased in mean attack frequency (14–17). For those studies combined, a total of 123/165 (70%) of patients were responders (≥50% decreased in mean attack frequency) and 74/114 (65%) were long term responders per this review definition. Refer to Supplementary Table S1 for a list of all primary outcomes. Two studies employed unilateral lead placement (one of these was strictly unilateral) (16, 18). All used continuous stimulation but patients were allowed to turn ONS on and off.

The decreased use of preventative medications was variable post-ONS for cluster headache. Leplus et al. defined as resistance to adequate trials including verapamil up to 960 mg/d, lithium with plasma level from 0.6 to 1 mEq/l and association of both, in absence of adverse events. In this study, preventative medications were reduced or stopped in 41.9% of all patients (15). Leone et al. selected for chronic cluster headache in the past year and treatment resistant to all available prophylactic medications for cluster headache (minimum of 10 medications) (14). All needed to maintain prophylaxis during ONS treatment. In Miller et al., twenty seven from the total cohort took preventative medications at baseline, and from those, 4 stopped altogether during the trial and 17 were able to deescalate preventative therapy (17). In Magis et al. eight patients out of 10 continued with preventative medication use (16). In Burns 2007 et al., three patients were taking a preventative (verapamil) prior to ONS. Post-implantation, one patient stopped due to side effects of this medication, the others continued with preventative verapamil use. There was no patient who initiated preventative therapy during the Burns study (19).

Not all studies evaluated use of abortives or reported decreases in those medications. In Leplus et al. abortive medications were reduced from 15 sumatriptan injections per week on average to 3 injections per week at last follow up. Oxygen consumption was reduced from 10 uses per week to 3.5 uses per week (15). In Leone et al. medication overuse headache was not present in patients prior to implantation (14). In Burns 2007 et al. no patient was pain free.However, one stopped using triptans,three reduced use of triptans, and 4 did not change their use (19). In Burns 2009 et al., there was only one patient who used intermittent dexamethasone as a transitional treatment for cluster headache attacks, but others without any additional new medications added (20).

Habituation: There was evidence of a loss of efficacy over time in 2 studies (14–16). For cluster headache only two studies had both short-term and long-term clinical outcomes providing insight regarding the possibility of a habituation response to ONS. In the Leplus et al. study, there were 7/42 patients (17%) who had lost the initial effectiveness of ONS in long term follow up and so the effects of habituation are minimal in this treatment resistant population based on the data from this study. In the Leone et al. study, five patients (out of 20) had originally shown a reduction of ≥50% reduction of headaches per day, which lasted for an average of 14.6 months (range 2–48 months). It was speculated that this could have been due to the natural course of the disease vs. “tolerance” AKA habituation response. Potentially 25% of patients had loss of efficacy which could be related to habituation. From the Magis et al. study, response to ONS was sustained over a mean period of 71 months.

Adverse events: Studies reporting adverse events listed dates of ONS implantation from 2002 to 2019. There were a total of 202 events in 153 patients out of a total of 245 patients in the 7 studies including cluster headaches in their cohort. It should be noted that these were total patients in the study and in some studies other headaches such as occipital neuralgia and migraine were included in the cohorts.

For the 6 studies restricted to cluster headaches, a total of 188 events in 143 patients out of a total of 228 patients.

### Migraine

Long-term clinical outcomes: There was a total of 105 patients with chronic migraine out of 5 studies combined (18, 21–24). Primary outcomes differed from study to study.Primary outcomes varied in that they assessed for a change in pain frequency or severity in the cases of Palmisani and Rodrigo or an assessment only for change in pain severity alone as in Harland and Miller as well as in the percentage of change ((≥30% in Miller vs. ≥ 50% in Harland) (21–24). Please refer to Supplementary Table S1 for a description of each study's primary outcome and results. Three studies employed both unilateral and bilateral lead stimulation (18, 23, 24). Two studies did not specify whether stimulation was continuous or intermittent (23, 24). Two studies investigated using continuous stimulation (18, 21).

In the Roderigo and Miller studies, 70% and 20% reductions in analgesic use were reported respectively. In the Roderigo et al. study, patients were able to continue all abortive medications and 14/35 (40%) of patients were not taking any analgesic medications on final follow up (22). Acute medications in Miller et al. were reported for the entire cohort and acute medication use fell by 2.43 days, a reduction of 20% which was not statistically significant (21).

Preventative medications were variably utilized in each study. In the Roderigo et al. study, patients were able to continue preventatives during the study and the average number of concomitant drugs was reduced from 4.4+/−1.7 at baseline to 1.3+/−1.6 on last follow-up visit (22). In the Miller et al. study, patients who were refractory to adequate trials of at least 3 preventative drugs per the European Headache Federation guidelines were offered ONS (25). Medications were changed as needed during the study. A mean of 9.36 (±2.61) preventative medications had been trialed before ONS. Twenty-three patients from the entire cohort were taking preventative medications prior to ONS. After ONS, 6 had stopped taking any medications, 4 had reduced the dose or stopped at least one medication, 8 had no change to the medication dosage and 5 had increased the number of medications (21). In the Palmisani et al. study, patients had failed adequate trials of 4 classes of preventatives and 3 classes of acute drugs prior to ONS, however no description of continuation on preventatives throughout the study was described (24). In Brewer et al. a mean of 11 (range was 6−19) migraine preventatives were trialed prior to ONS for treatment of migraine alone.This number did not include Onabotulinum toxin A which was used in 7 patients (18).

Medication overuse was not addressed in 3 studies (Palmisani, Harland, and Brewer et al.). Roderigo et al. excluded patients overusing acute medications. In Miller et al., twenty patients were overusing acute medications at time of implant, but these values represent the entire cohort, including patients with additional headache types. All patients with chronic migraine alone and medication overuse had undergone a withdrawal period from overuse and failed to report any improvement (21). Per Palmisani et al., not enough data was collected to comment on whether medication overuse headache was contributing (24).

Only Roderigo excluded for no response to diagnostic nerve block (22). Miller et al. was the only study which did not employ trial stimulation prior to permanent placement (21). In Palmisani's study, one patient decided to proceed despite a negative stimulator trial and had a mild benefit (<50% relief in headache frequency or intensity, further distinction was not specified in the study) after 5 years of follow up (24).

No habituation response was suggested by the Roderigo and Miller studies.

Adverse events: Studies reporting adverse events listed dates of ONS implantation from 2002 to 2019. There were a total of 72 events in 48 patients out of a total of 124 patients in the 5 studies including migraine headaches in their cohort. It should be noted that these were total patients in the study and in some studies other headaches such as occipital neuralgia or cluster headaches were included in the cohorts.

For the 3 studies restricted to migraine, a total of 62 events in 38 patients out of a total of 107 patients.

### Occipital neuralgia

Long-term clinical outcomes: There were 6 studies that included patients with greater than 24 months of follow up when occipital neuralgia was the indication for ONS. Note that the Johnstone study did not provide duration of individual patient responses and mean follow up evaluation was 25 months, so it is unclear which how many patients sustained long term benefit as defined by this narrative review from Johnstone et al. From studies which did outline patient follow up after 24 months for occipital neuralgia, there were a total of 41 patients in the studies combined, with 5 patients responding long term with decrease in numeric rating scale (NRS) and 14 patients responding with decrease in visual analog scale (VAS). Outcome measures for all studies incorporated pain severity. Harland et al. reviewed decrease in severity on NRS. Slavin et al., Johnstone et al. and Magown et al. reviewed decrease in severity on VAS (18, 23, 26–28). Ultimately, outcome success in both Brewer et al. and Weiner et al. was determined by patient qualitative description of benefit (18, 29). See Supplementary Table S1 for outcome measures. All studies except for Magown included patients with both unilateral and bilateral leads and varied in continuous vs. intermittent stimulation (18, 23, 26, 27, 29). In the Brewer et al., lead laterality was not specified for occipital neuralgia specifically, but noted that for unilateral headaches, unilateral leads were used and for bilateral headaches, bilateral leads were employed. Lead laterality was not discussed in the Magown study. Brewer employed continuous ONS and Slavin et al. allowed for patients to turn the device off and use intermittently (18, 26). Magown also employed intermittent stimulation (28). The stimulation schedule was not discussed in the Harland study (23).

In Slavin's study, patients continued to use non-opioid analgesics for pain control throughout the trial and it was noted that 3 continued with mild abortives and one patient required strong non-opioid analgesics on last follow up (26). All patients except 2 in the Magown study stopped all pain medications (28). All studies except for Brewer and Magown trialed patients with occipital nerve blocks prior to ONS. Magown trialed C2 blocks. For Slavin, all patients were trialed with nerve stimulation prior to permanent ONS and 10/14 went to have permanent implantation (26). In Harland's study, 7 patients were responsive to occipital nerve block prior to permanent ONS. All patients in Harland, Brewer and Weiner studies were trialed with neurostimulation prior to permanent stimulation implantation surgery (18, 23, 29). Eight patients in the Johnstone study were trialed with peripheral nerve stimulation and the one patient which did not achieve benefit from trial stimulation was excluded (27).

Habituation could not be determined from the data presented in any study except for Slavin et al. as no post-implantation follow up was available other than the long-term results. There were a limited number of patients in the Slavin study who were observed to be responders at 6 months and maintained response at greater than 24 months (*n* = 4).

Adverse events: Studies reporting adverse events listed dates of ONS implantation from 1999 to 2019. There were a total of 17 events in 34 patients out of a total of 54 patients in the 5 studies including occipital neuralgia in their cohort. It should be noted that these were total patients in the study and in some studies other headaches such as migraine or cluster headaches were included in the cohorts.

For the 4 studies restricted to migraine, a total of 7 events in 29 patients out of a total of 37 patients.

### Cervicogenic headache

Long-term clinical outcomes: Sixteen patients were included in the Eghtesadi et al. study and included if they had a strictly unilateral headache without side shift, moderate to severe intensity for at least 4 h per day, more than 15 headache days per month, and present for over a year (30). Six patients with a combination of different headache types (migraine and cluster) and cervicogenic headache were included. Median duration was 15.0 years and all patients suffered from daily cervicogenic headaches at baseline assessment. The primary outcome was reduction in headache frequency and assessments occurred at one- and three-years post-implantation. Patient achieving a 50% or greater reduction in headache frequency were considered responders. At one year, 11 patients (69%) were responders (exhibited greater than 50% reduction headache frequency). See outcomes in Supplementary Table S1. Unilateral leads were placed. There was no description of the schedule of stimulation (intermittent vs. continuous).

Patients were each refractory to 4 or more preventive medications. Patients previously trialed occipital blocks, C2–3 intraarticular facet joint injections, C2–3 medial branch blocks and radiofrequency ablation. In patients with comorbid chronic migraine, Botox following a PREEMPT protocol was also trialed. Medications were not modified for one month prior to permanent implantation and medication modifications were permitted 3 months after permanent ONS was implanted. There was medication overuse with triptans in 6 patients and narcotic overuse in 6 patients at baseline assessment.

Habituation: At the 3-year follow-up, 6 patients were responders defined as sustaining ≥50% reduction in headache frequency. Four patients among these reverted to episodic frequency from 5 prior responders at 1 year and 1 new responder since the 1-year assessment. Six of the patients who were noted to be responders at 1 year lost response beyond 1 year, and of these 3 had coexistent migraine. Of the 10 patients with only cervicogenic headache at 1 year, 7 were originally responders.At 3 years, only 4 patients, including a patient who became a responder after 1 year of no response, were responders. This outcome suggests a habituation response in a limited subset of patients. There were no differences in terms of abortive medications from responders to non-responders. Work status related to habituation could not be assessed from the data presented. Five patients out of 7 on work disability at baseline returned to work at the 3 year timepoint assessment.

Adverse events: Eghtesadi et al. included patients implanted between 2011 and 2013 and 13 out of 16 patients had adverse events.

### Short-lasting unilateral neuralgiform headache attacks (SUNHA)

Long-term clinical outcomes: Miller et al. performed an uncontrolled open-label prospective study evaluating long term ONS treatment outcomes including or beyond 24 months in 31 patients with SUNHA (out of which 15 did not have overlapping other headache types) (31). Primary endpoint was the change in mean daily attack frequency at ultimate follow up. Successful responders were defined as having improvement of at least 50% or greater. See Supplementary Table S1 for outcomes. Bilateral leads were implanted in every patient and goal was to implement a continuous stimulation schedule.

Patients had failed adequate trials of 7 different preventative medications on average. Indomethacin was also trialed in patients with longer lasting attacks to determine whether these were indomethacin responsive headaches. In all patients in the study (*n* = 31), there was a 24% reduction in the number of patients taking preventative medications on follow up. Only 3 patients had medications started for SUNHA during ONS (in responders). Medication overuse was not discussed.

Patients were not trialed with either occipital nerve blocks or trial stimulation.

Habituation: Could not be determined from the information provided.

Adverse events: Miller et al. included patients implanted from 2007 to 2015 and reported 25 events in 20 patients out of a total of 31.

### Paroxysmal hemicrania

Long-term clinical outcomes: One case report described sustained efficacy of ONS with more than 50% reduction in mean attack frequency in a female patient followed up to 120 months (32).

Severe gastric side effects despite proton pump inhibitors precluded continuation of indomethacin at an effective dose. Prior to ONS, trials of 9 other preventatives failed. Occipital nerve blocks were ineffective. There was no report of stimulation trial performed prior to permanent implantation, however the study does refer to a prior migraine study for details of ONS implantation and in that study no trial was employed (21).

Ultimately, the patient obtained satisfactory pain control off indomethacin with ONS. Benefit continued through 3 pregnancies. Bilateral leads were placed, and stimulation was continuous.

Habituation: This case report does not support evidence of habituation.

Adverse events: One event in one patient was reported in the Miller et al. study and implantation was performed in 2006.

## Discussion

Studies to date on long-term ONS outcomes in headache populations intractable to conservative medical management have shown long term benefit when the devices are functioning properly. However, these prior long-term outcome studies were complicated by high rates of adverse events including lead migration which makes the assessment of habituation difficult. In the past, spinal cord stimulator leads were used “off-label” for ONS. More recently, there have been stimulator leads designed specifically for peripheral nerve stimulation that connect to an external pulse generator applied to the skin with at least one device obtaining FDA clearance for treatment of headache (33–35). The long-term outcomes with the new peripheral nerve stimulator devices in ONS are not known due to only recent FDA clearance for use, but the efficacy observed with the traditional ONS approach is likely translatable to the new FDA cleared peripheral nerve stimulator devices which have much lower rates of technically associated adverse events since the newer devices are specifically designed for peripheral nerve stimulation. With the paucity of data, we were unable to perform a systematic review, but tried to derive some conclusions from the data available in our narrative review.

Despite the high rate of adverse events, clinical outcomes were improved overall. For all studies, at any follow up or 1 year follow ups, the cumulative number of responders (as defined per each individual study) to ONS was 264/397 (66%). For all studies except Johnstone, at the longest term follow up (mean equal to or greater than 24 months) the cumulative number of responders to ONS was 177/311 (56%). Results from the Johnstone study were not included in this number as the mean follow up was 25 months and the specific duration of follow up was not provided. Overall, there was a paucity of literature assessing outcomes at 2 separate time points in order to make a distinction of loss of efficacy over time. The available literature providing this information was limited to patients presenting with cluster headache and cervicogenic headache. In the cluster headache population, only a minority of patients had a loss of efficacy. Typically, in chronic cluster headache, it takes time for ONS to take effect. Once effective, the patients in the two long term studies, Leplus et al. and Leone et al., continued to observe benefit in long term follow-up with 17%–25% of patients having a loss of efficacy after mean 24 months of treatment who initially demonstrated a treatment response. Specifically, it is difficult to assess habituation in the chronic cluster headache population due to the inherent fluctuations that occur with the natural history of the disorder. In the Magis study, 40% of patients followed long term (mean 71 months) were observed to have experienced a change from chronic to episodic cluster headache which was likely related to ONS but could have been related to the natural history of cluster headache (16). However with the natural history of cluster headache there is a conversion to episodic cluster headache only in a minority of cases (7.4 to 33%) and in the natural history of chronic cluster headache the majority of patients reported no change in headache frequency and/or severity (60%) (36–38). In the single study of patients with cervicogenic headache where initial response and long term response were both documented, there was a higher rate of loss of efficacy in a sample of patients having dual diagnoses (migraine and cervicogenic headache) which may represent a cohort of patients with more treatment resistant headaches (30). With the studies available, we were unable to make any meaningful assessments on loss of efficacy or habituation in occipital neuralgia, chronic migraine, SUNHA and paroxysmal hemicrania populations.

There is variability and inconsistencies in reporting of analgesics, medication overuse, habituation, and outcomes. There are no consensus criteria for habituation in neuromodulation and this will be needed for future study of habituation. The variability in reporting in the studies for occipital nerve stimulation was in part related to not having guidelines for reporting as a reference when the studies were implemented. The prevalence of medication overuse headache was not well defined in the studies and this may have had a negative effect on outcomes which has been demonstrated in other treatment trials in migraine (39, 40). Given this, it is difficult to ascertain if any decreases in long term efficacy were due to perceived habituation or the presence of a confounder like medication overuse headache. Future studies of occipital nerve stimulation should follow reporting as recommended in the guidelines for controlled trials of preventive treatment of chronic migraine (41).

In review of long term effectiveness for chronic migraine compared to Chen's systemic review which defined long term as greater than 1 year, our review provides support for maintenance of effectiveness in patients at 24 months (42). Yang's systemic review defined long-term follow up as 1.5 years and they noted similar variability in outcome measures and definition of response rate among studies (43). This review did not specifically focus on long-term outcomes but did note that mean number of headache days in most studies were decreased and that only a small number achieved 50% improvement in severity which is the benchmark typically used for responder rates (22). Our review showed a high frequency of adverse events for migraine that was similar to the prior report of the Yang et al.

Overall, we found in our review that for long-term outcomes, the majority of patients which continued evaluation beyond 24 months had benefited from ≥50% of pain relief on either VAS or NRS. The findings of this review further support the recommendation by Sweet al. for use of ONS in treatment of medically intractable occipital neuralgia (44).

Long-term outcomes did not appear to be predicted by a positive occipital nerve block or stimulator trial. Newer devices can be implanted for up to 60 days for a trial and a paradigm shift is occurring in peripheral nerve stimulation where some studies show that the duration of effect for peripheral nerve stimulation lasts longer than the duration of treatment (45–48). The Magis study of cluster headache showed improved outcomes with the device turned off resulting in explant in one patient and there is the potential for occipital nerve stimulation to be used as a transitional treatment in medically refractory cluster headache (16).

The definition of “medically intractable” varied in the studies with some having specific number of treatments failed and others determined by the evaluating physician. The definition of “medically intractable” is changing with the development of new FDA approved treatments such as onabotulinum toxin A injections following a PREEMPT protocol and CGRP monoclonal antibodies. In clinical practice the use of these treatments in conjunction with ONS may occur or the use of an implantable devices may not be needed in patients who responded to these less invasive treatments (18, 24). Patient selection is a key factor that influences long term outcomes. In the right clinical context such as in the case of chronic cluster headache, ONS may provide a therapeutic option for patients who otherwise may not have alternative options.
