# Supplementary material for: Long term outcomes of occipital nerve stimulation
Source: Front Pain Res (Lausanne). 2023 Mar 20;4:1054764. doi: 10.3389/fpain.2023.1054764 (PMC10067723; doi:10.3389/fpain.2023.1054764)
Supplement: Supplementary file 1 [file Table1.docx]

**Table 1: Outcomes of Occipital Nerve Stimulation by Headache type**

**Cluster Headache**

| **References** | **Study type** | **Duration of study – median or mean/range if available (months)** | **Number of patients after pre-intervention (N)** | **Outcome parameters** | **Outcomes reported at time of follow up** | | |
| --- | --- | --- | --- | --- | --- | --- | --- |
|  |  |  |  |  | **Pre-intervention** | **Initial follow up post** | **Long-term follow up≥24 mo** |
| Brewer et al. | Retrospective review | Median: 72/  5-102 | 5 | Success of ONS as deemed by the investigator and the patient | All trialed on subcutaneous ONS 3-7 days and obtained ≥50% pain relief | 4/5 success per investigator, 3 unknown whether patient would repeat the procedure at final follow up | 2/5 with ≥50% benefit |
| Burns et al. (2007) | Case series | Median or mean not given/ 8-27 | 8 | To report long term follow up in ONS in patients with medically intractable chronic cluster headache | None | Useful effect built up over months, never in less than weeks | 6/8 noted improvement |
| Burns et al. (2009) | Case series | Median: 17.5 mo/4-35 mo | 14 | Describe clinical outcomes of ONS in patients with medically intractable chronic cluster headache | None | No interim evaluation | 10/14 patients improved (3 by 90%, 3 by 40%, 4 by 20-30%) |
| Leplus et al. | Prospective study | Mean:44 mo/19-106 | Out of 105, 93 completed any follow up, 44 continued =1 yr follow up (42 for long term follow up >1 yr) | To evaluate sustainability of ONS efficacy over time and identify predictive factors of efficacy | None | 64/93 with follow up of any duration had mean attack frequency decrease ≥50%  At 1 yr, 44 patients assessed with a 75% responder rate | Out of 42 who went on for long term evaluation, 28 were long term responders (mean 4 yrs), 7 lost initial effect and 2 lost to follow up. 4 previously non-responsive at 1 year became responsive after resolution of technical issues |
| Leone et al. | Monocentric experimental open label study | Median:73/19-128 | 35 (only 30 with follow up) | Reduction in the number of headache daily attacks and responders were 50% reduction in frequency or greater | None | At one year, 25/30 patients were responders | After median follow up of 6 years, 20/30 (66.7%) were responders  At median 6 years follow up, median # of daily attacks dropped from 5.7 to 2.4 |
| Magis et al. | Prospective pilot study | Median: 61/4-103 | 15 | Discuss the very long term outcomes of a drug refractory chronic cluster headache management | None | Mid-term follow up (avg 36 mo), 10/15 retained implants, all pain free | Mean 71 months, 4 patients, attacks relapsed in an episodic pattern, other 6 became chronic again, reduction of 70.8% of mean attack frequency |
| Miller et al. | Prospective study | Mean: 45/2-81 | 32/51 (chronic cluster alone) | Improvement in mean daily attack frequency at final follow up compared to baseline. Patients with 50% or more reduction in mean daily attack frequency were considered responsive to treatment | None | None | 17/32 (53.1%) with a 50% response in CCH at final followup  Mean daily attack frequency was reduced by 49.5% (+/-43.84%) at final followup |

**Migraine Headache**

| **References** | **Study type** | **Duration of study – median or mean/range if available (months)** | **Number of patients after pre-intervention (N)** | **Outcome parameters** | **Outcomes reported at time of follow up** | | |
| --- | --- | --- | --- | --- | --- | --- | --- |
|  |  |  |  |  | **Pre-intervention** | **Initial follow up post** | **Long-term follow up≥24 mo** |
| Brewer et al. | Retrospective review | Median: 61/1-70 (migraine only) | 12 migraine, 10 with only migraine, 5 who were followed for ≥24 months | Success of ONS as deemed by the investigator and the patient | All trialed on sub-cutaneous ONS 3-7 days and obtained ≥50% pain relief | None | 5 patients; 3 who would repeat, 2 unknown; successful per investigator: 3/5 |
| Harland et al. | Retrospective review | Median: 31.5/8-63 | 6 total, 4 without concurrent supraorbital stimulation | Responders to ONS had a reduction of pain severity by 50% on NRS by final follow up | All trialed on PNS and obtained ≥50% pain relief | None | 2 patients were responders, 2 were not |
| Palmisani et al. | Retrospective review | 5 subjects duration unknown, of those known: Median:180/12-228 | 19 (17 actually implanted) | Success of ONS determined by a continued reduction of at least 50% in headache intensity and/or frequency reported by the patient on phone interview, associated with decreased medication use | All trialed with per-cutaneous lead trial for 7-10 days and obtained ≥ 50% pain relief | 9/17 deemed successful at final follow up | 5/9 successful with follow up ≥24 months |
| Roderigo et al. | Uncontrolled open label prospective observational study | Mean:108±72;  31 patients with 7 yr follow up | 37 | Decrease in headache frequency by ≥50% from baseline and/or ≥50% decrease in VAS | All trialed for 10 days post-sub-cutaneous implant, and >50% decrease in VAS selected for perm implant | 31/35 (89%) with greater than 50% reduction in headache frequency | 30 patients with decreased VAS by 3.8 +/- 2.5 points, 5 patients remained pain free |
| Miller et al. | Uncontrolled, open label prospective study | Median:42/6-97 | 53 total and 35 CM only | Decrease in mean monthly moderate-to-severe headache days at final follow-up by with 30% or greater reduction compared to baseline. | None | None | 30% or more reduction in moderate to severe headaches was observed in 36.4% (n=12) at final followup; mean daily pain score was -1.20 points in VRS |

**Occipital Neuralgia**

| **References** | **Study type** | **Duration of study – median or mean/range (months)** | **Number of patients after pre-intervention (N)** | **Outcome parameters** | **Outcomes reported at time of follow up** | | |
| --- | --- | --- | --- | --- | --- | --- | --- |
|  |  |  |  |  | **Pre-intervention** | **Initial follow up post** | **Long-term follow up≥24 mo** |
| Brewer et al. | Retrospective review | 9-90 (occipital neuralgia only) | 2, 1 who met criteria of follow up | Overall benefit and would the patient repeat | All trialed on subcutaneous ONS 3-7 days and obtained ≥50% pain relief | None | 90 months, pain spontaneously resolved, stimulator not in use; 9 months, 30% difference and would not repeat |
| Harland et al. | Retrospective review | Median: 45/1-79 | 9 | Responders had 50% reduction on NRS as a marker of response | All trialed on PNS and obtained ≥50% pain relief | None | 5/7 continued responders |
| Slavin et al. | Retrospective review | Mean: 22/5-32 | 10 | Greater than ≥50% pain relief as evidenced by VAS prior to surgery, trial and at first and last follow up, analgesic use before and after surgery and degree of patient satisfaction with pain relief | All patients responded positively to occipital nerve blocks with temporary relief and successful trial of PNS with ≥50% pain relief for 5-7 days | 10/10 responders for at least 6 months | 6/10 total continued follow up at **≥**24 mo, 4/10 with ONS still implanted and 60-90% pain relief, 2 removed: 1 due to pain free without stimulation and 1 due to either loss of effect or infection (not specified to this level of detail) |
| Weiner et al. | Retrospective observational study | Mean:29/18-66 | 13; 6 with follow up ≥24 mo | Greater than ≥50% pain relief from patient description of response | All patients responded positively to occipital nerve blocks with temporary relief and had trial of 5-7 day stimulation | None | 6 continued follow up at ≥24 mo, 1 with resolved symptoms and an explant, 3 with >75% improved pain relief and 2 with >50% improved pain relief |
| Johnstone et al. | Case series | Mean:25/6-47 | 7 | Greater than ≥50% pain severity reduction in VAS, medication reduction, and activities of daily living | 7 patients included responded positively to occipital blocks and trial of occipital stimulation for 1 week with >50% pain reduction | None | At mean final follow up of 25 mo (for all patients), 2 patients ceased all medications/procedures;  Reduction in VAS in 5/7. Two acquired full time employment. |
| Magown et al. | Case series | Mean:18/2-30 | 7; 3 with follow up ≥24 mo | Description of surgical technique and outcomes in terms of pain severity reduction on VAS | All patients responded to double blinded C2 nerve root blockade | None | At mean final follow up of 18 mo in 4 patients, mean pain severity reduction of 96% on VAS; in the 3 with final follow up at ≥24 mo with 2/3 with pain resolution on PNS and 1/3 with 75% pain reduction |

**Cervicogenic Headache**

| **References** | **Study type** | **Duration of study – median or mean/range if available (months)** | **Number of patients after pre-intervention (N)** | **Outcome parameters** | **Outcomes reported at time of follow up** | | |
| --- | --- | --- | --- | --- | --- | --- | --- |
|  |  |  |  |  | **Pre-intervention** | **Initial follow up post** | **Long-term follow up≥24 mo** |
| Eghtesadi et al. | Retrospective review | 36 months duration | 16 (10 with cervicogenic headache alone) | 50% decrease in headache frequency | All patients have daily headaches at baseline assessment | At one year, 11/16 patients showed more than 50% reduction in headache frequency | At 3 years, 6/16 were responders. 6 lost response and one gained response |

**SUNHA**

| **References** | **Study type** | **Duration of study – median or mean/range if available (months)** | **Number of patients after pre-intervention (N)** | **Outcome parameters** | **Outcomes reported at time of follow up** | | |
| --- | --- | --- | --- | --- | --- | --- | --- |
|  |  |  |  |  | **Pre-intervention** | **Initial follow up post** | **Long-term follow up≥24 mo** |
| Miller et al. | Uncontrolled open-label prospective study | Mean :44.2 /13-81 | 31 (15 with SUNHA alone) | 50% or greater reduction in daily attack frequency | All patients have daily headaches at baseline assessment | Mean time for all patients (n=31) to reach 50% improvement was 5.7 mo (range 1-19) | For SUNHA alone, 67% (n=10) with ≥50% reduction in daily attack frequency at final follow up |

**Paroxysmal Hemicrania**

| **References** | **Study type** | **Duration of study – median or mean/range if available (months)** | **Number of patients after pre-intervention 30 (N)** | **Outcome parameters** | **Outcomes reported at time of follow up** | | |
| --- | --- | --- | --- | --- | --- | --- | --- |
|  |  |  |  |  | **Pre-intervention** | **Initial follow up post** | **Long-term follow up≥24 mo** |
| Miller et al. | Case report | 120 months | 1 (with coexistent migraine) | 50% or greater reduction in daily attack frequency | 6-8 attacks daily lasting 5 to 60 min, mean of 15 min | 1 month post- ONS: attacks were occurring once per week, no change to severity or duration from pre-ONS | Pain free at 120 mo follow up |

**Table 2: Adverse Events**

| **Study name (year of publication): years of trial** | **Headache type (s)** | **Reaction (including patients with less than 24 months follow up in all categories)** |
| --- | --- | --- |
| Brewer et al.(2012):2002-2011 | Cluster/migraine/occipital neuralgia | 10/17 patients:13 lead revision procedures in 10 patients; 1 of these with infection |
| Burns et al. (2007):not included | Cluster | 7 in 4/8 patients: Needing new electrode and leads, lead migration (n=4), battery malfunction (n=1) excessive pain at scar site post-op (n=1), shock like sensation (wire kinking) (n=1) |
| Burns et al. (2009):2003-2006 | Cluster | 20 in 12/14 patients: discomfort or pain/erythema/sensitivity at scar site (n=8), electrode migration/failure/additional needed (n=6), wires kinking (n=1), overstimulation or varying stimulation (n=2), lead discomfort (n=1), painful neck movements (n=2) |
| Leplus et al.(2020): not included | Cluster | 80 in 67/105 patients: infections (n=8), lead migration (n=16), lead fracture (n =6), insufficient perception of paresthesias (n=10), wound issue (n=2), hardware dysfunction (n=11), local pain at the lead (n=8) or generator (n=19) |
| Leone et al (2017):2004-2014 | Cluster | 11 in 10/35 patients: 8 with electrode migration and malfunctions. 3 wire migrations and malfunctions. |
| Magis et al.(2016):2005-2009 | Cluster | 8/15; (53%) Infection 1/15, delayed infection 3/15, unbearable paresthesias 2/15, lead externalization/migration 2/15 |
| Miller et al.(2016):2007-2014 | Cluster (entire cohort, including mixed headaches as not individually outlined in study) | 62 events affecting 35/51 patients: Most common others: pain over IPG lead/wound sites (n=12 (23.5%)) and undesirable changes in stimulation (n=17 (33.3%)) |
| Harland et al.(2020): 2012-2019 | Migraine/Occipital neuralgia | Not listed |
| Palmisani et al. (2013) 2007-2012 | Migraine | 3/17 (18%) Painful paresthesia, battery site hyperalgesia, granuloma and skin erosion, inefficacy |
| Roderigo et al.(2017): 2002-2013 | Migraine | 9/37 (24%) Painful stimulation (n=2), lead externalization (n=4), infection (n=3) |
| Miller et al. (2016):2007-2013 | Migraine (entire cohort, including mixed headaches as not individually outlined in study) | 50 events in 26/53 patients: Included electrode erosion and system revision, neck stiffness and pain over wound sites, stimulation undesirable changes in stimulation, allergy to surgical material and infection |
| Magown et al. (2009): not included | Occipital neuralgia | 1/7 (14%): 1 case of seroma with wound dehiscence |
| Johnstone et al. (2006): 2001-2004 | Occipital neuralgia | 2/7 (29%) 2 cases of infection |
| Slavin et al.(2006): not included | Occipital neuralgia | 3 events in 2/10 patients: 1 infection, 1 electrode migration, tightness in neck and spasms in the neck and right hemibody |
| Weiner et al. (1999):not included | Occipital neuralgia | 1/13 (8%) 1 case of lead migration |
| Eghtesadi et al.(2018): 2011-2013 | Cervicogenic | 3/16 (19%) 3 cases (Infection, discomfort at implant site, migration) |
| Miller et al.(2018):2007-2015 | SUNHA | 25 events in 20/31 patients: 1 infection, 7 pain over wound sites, 7 neck stiffness, 1 with allergy to surgical material, 9 undesirable changes in stimulation |
| Miller et al.(2017):2006 | Paroxysmal hemicrania | 1 event in 1 patient: Unequal pain over the electrode |

**Table 3: Habituation response**

| **Study name** | **Headache type (s)** | **Total number of responders at 1 -year follow up** | **Total number of responders at long term follow up (mean ≥24 months)** | **Number of patients with loss of efficacy** |
| --- | --- | --- | --- | --- |
| Leplus et al. | Cluster | 33/44 | 28/42 | There were 7/42 patients who had lost the initial effectiveness of ONS at mean follow up of 4.1 years |
| Leone et al. | Cluster | 25/30 | 20/30 | 5/30 patients originally shown a reduction of ≥50% reduction of headaches per day, which lasted for an average of 14.6 months (range 2-48 months). In 4, improvement lasted up to 12 mo, and in remaining pt, improvement lasted 4 years |
| Eghtesadi et al. | Cervicogenic | 11/16 | 6/16 | 6/16 lost response |
